# Surgical Conversion for Initially Unresectable Locally Advanced Hepatocellular Carcinoma Using a Triple Combination of Angiogenesis Inhibitors, Anti-PD-1 Antibodies, and Hepatic Arterial Infusion Chemotherapy: A Retrospective Study

**DOI:** 10.3389/fonc.2021.729764

**Published:** 2021-11-12

**Authors:** Jinliang Zhang, Xihao Zhang, Han Mu, Ge Yu, Wenge Xing, Lu Wang, Ti Zhang

**Affiliations:** ^1^ Key Laboratory of Cancer Prevention and Therapy, Tianjin Medical University Cancer Institute and Hospital, Department of Hepatobiliary Surgery, National Clinical Research Center for Cancer, Tianjin, China; ^2^ Key Laboratory of Cancer Prevention and Therapy, Tianjin Medical University Cancer Institute and Hospital, Department of Interventional Therapy, National Clinical Research Center for Cancer, Tianjin, China; ^3^ Department of Hepatic Surgery, Fudan University Shanghai Cancer Center, Shanghai, China; ^4^ Department of Oncology, Shanghai Medical College, Fudan University, Shanghai, China

**Keywords:** advanced hepatocellular carcinoma, combination therapy, hepatectomy, conversion therapy, hepatic arterial infusion chemotherapy, anti-PD-1, China

## Abstract

**Background:**

Recent research has shown that selected patients with initially unresectable hepatocellular carcinoma (HCC) are able to achieve conversion to resectable disease through systemic or local therapy. Combination regimens comprised of drugs with different mechanisms of action have shown better outcomes than single-drug or single-approach-based treatments; however, to date, combination regimens investigated as part of conversion therapy strategies have been two drug combinations with reported issues of relatively low surgical conversion and objective response rates. In this study, we investigated the efficacy and safety of triple combination therapy with angiogenesis inhibitors, programmed death-1 inhibitors and hepatic arterial infusion chemotherapy for surgical conversion of advanced HCC.

**Methods:**

This was a single-center, retrospective, single-arm study of patients with unresectable HCC who received at least one cycle of triple combination therapy with an oral anti-angiogenic drug, programmed death-1 inhibitors and hepatic arterial infusion chemotherapy between August 2019 and August 2020. Endpoints included the overall response rate (ORR), surgical conversion rate, time to response and safety. Treatment response was assessed using the modified Response Evaluation Criteria in Solid Tumors (mRECIST) and RECIST v1.1.

**Results:**

In total, 34 patients were included in this study, of whom 25 completed treatment evaluation. The best ORR was 96.0% (24/25); 48.0% (n = 12) had a complete response, 48.0% (n = 12) had a partial response, and 4.0% (n = 1) had stable disease. The median time to response was 50.5 (95% CI, 31.02–64.00) days and the surgical conversion rate was 60% (15/25). Of the 25 patients, 56.0% (n = 14) received surgical resection and 28.0% (n = 7) had a pathologic complete response. Toxic side effects were manageable.

**Conclusion:**

A triple combination therapy regimen of angiogenesis inhibitors, programmed death-1 inhibitors and hepatic arterial infusion chemotherapy showed significant therapeutic effect with an extremely high surgical conversion rate in patients with initially unresectable HCC.

## Introduction

Hepatocellular carcinoma (HCC) is the sixth-most-common cancer and the third leading cause of cancer-related deaths worldwide (1). Owing to the absence of symptoms in the early stages of the disease, more than 70% of patients with HCC are diagnosed at an advanced stage, long after transplantation, surgery or locoregional treatment are feasible (2). Palliative systemic therapy is usually the only remaining treatment option for these patients. However, in the past few years, treatment of advanced HCC has evolved rapidly with the introduction of novel systemic therapies.

Although the mechanisms of action of new therapies for HCC are not fully understood, therapeutic regimens that combine drugs with different mechanisms of action have shown significantly better outcomes than single-drug or single-approach-based treatments. Combination regimens that have been investigated include two-drug combinations such as two immune checkpoint inhibitors, immune checkpoint inhibitors with molecular-targeted drugs or sequential use of two molecularly-targeted drugs (3–10); immune checkpoint inhibitors with locoregional treatment (4, 11); and molecularly-targeted drugs with locoregional treatment (12–14). The furthest advanced combination therapy to date is dual combination treatment with a tyrosine kinase inhibitor and immune checkpoint blockade (atezolizumab plus bevacizumab) which is a recommended first-line therapy for advanced HCC based on the phase 3 IMbrave150 trial in which it showed a significant survival benefit versus sorafenib (8).

In previous studies, hepatic arterial infusion chemotherapy (HAIC) demonstrated relatively high response rates (22–86%) and an acceptable toxicity profile (15, 16). In Japan, HAIC is considered a safe and effective alternative to sorafenib and is recommend for use in patients with advanced HCC (17). Recent clinical trials have also shown that sorafenib plus HAIC with oxaliplatin, fluorouracil, and leucovorin (FOLFOX) improved the objective response rate (ORR) versus sorafenib in patients with HCC and portal vein invasion; however, the survival benefit was unsatisfactory (18). Immune combination therapy is associated with the ‘survival drag effect’ in which patients with an effective immune response will achieve a highly durable antitumor response from a particular time point (the onset of the immune response) (19). In summary, the ORR of HAIC is high, but does not translate to an increase in OS. This can be compensated through use of a dual combination of a tyrosine kinase inhibitor and immune checkpoint blockade.

In clinical practice, selected patients with unresectable HCC can be converted to resectable disease through a variety of systemic or locoregional treatment strategies, and some studies have shown that salvage surgery following surgical conversion can achieve favorable outcomes in these patients. An effective way to improve OS with combination therapy is to proactively conduct radical treatments, such as conversion surgery or ablation (20). Indeed, salvage surgery following surgical conversion has been reported to achieve favorable outcomes in some studies (21). However, challenges reported with previously investigated approaches include relatively low objective response rates (ORRs) and surgical conversion rates, highlighting a need for the identification of more effective combination regimens with manageable side effects in order to allow a higher proportion of patients to achieve conversion to resectable disease. The present study investigated the efficacy and safety of a triple combination therapy strategy including angiogenesis inhibitors, programmed death-1 (PD-1) inhibitors and HAIC in patients with initially unresectable advanced HCC.

## Materials and Methods

### Study Design and Patients

This single-center, retrospective, single-arm study included patients aged ≥18 years with unresectable HCC confirmed by three independent hepatobiliary surgeons, with one or more measurable target lesions based on computerized tomography (CT) or magnetic resonance imaging (MRI) who had received triple therapy at Tianjin Medical University Cancer Institute and Hospital between August 1, 2019 and August 20, 2020. Eligible patients had not received previous treatment for HCC or had progressed on previous treatments, and had Barcelona Clinic Liver Cancer (BCLC) stage C disease, an A or B on the Child-Pugh liver function scale and an Eastern Cooperative Oncology Group Performance Status score of 0–2. Exclusion criteria included comorbidity with other severe systemic diseases, discontinuation of treatment for personal reasons or violating treatment procedures, and inability to tolerate or comply with treatment.

The study protocol was approved by the The Research Ethics Committee of Tianjin Medical University Cancer Institute and Hospital, which granted ethical approval for the use of human subjects (Approval No. bc2020007). The study was conducted in accordance with the Declaration of Helsinki and other ethical principles for medical research involving human subjects. All patients gave written informed consent before entering the study.

### Treatments

Patients were treated with a triple combination of angiogenesis inhibitors, anti-PD-1 antibodies, and HAIC. As long as a triple combination regimen was used, the brand of angiogenesis inhibitors and anti-PD-1 antibodies was not considered. Three kinds of angiogenesis inhibitors and two kinds of anti-PD-1 antibodies, which are commonly used in clinical practice, were used by patients in this study. To be more specific, eligible patients had received a triple combination of an oral anti-angiogenic drug (in order to reduce side effects and ensure curative effect, a low dose was used, based to the prescribing information: apatinib 250 mg/day, lenvatinib 8 mg/day, or sorafenib 400 mg twice daily), a PD-1 inhibitor (camrelizumab 200 mg or sintilimab 200 mg every 3 weeks) administered intravenously, and HAIC with FOLFOX administered every 4–8 weeks with the catheter and sheath removed at the end of infusion. The choice of anti-angiogenic agent was at the discretion of the patient. The FOLFOX regimen comprised oxaliplatin 85 mg/m^2^ as a 2 hours infusion, calcium folinate 400 mg/m^2^ as a 2–3 hours infusion and fluorouracil 400 mg/m^2^ as a bolus injection, followed by fluorouracil 1200 mg/m^2^ administered over 23 hours on day 1. HAIC dosing was adjusted appropriately according to the patient’s liver function with a minimum dosage of one third of the standard dose. Imaging evaluations (MRI was preferred and CT was used if MRI was not available) were conducted every 4-8 weeks after at least one HAIC treatment. Tumors were assessed every 2 months using serum markers and imaging examination with abdominal ultrasound as the primary method and contrast MRI on suspicion of recurrence. Patients determined to have sufficient residual liver volume after tumor resection, verified by senior surgeons during multidisciplinary meetings, received surgical intervention followed by oral angiogenesis inhibitors and PD-1 inhibitors.

### Measurements and Endpoints

Endpoints included ORR, defined as the percentage of patients with complete response (CR) or partial response (PR), time to response (TTR), progression-free survival (PFS), surgical conversion rate, 6-month PFS, 12-month PFS and safety. Safety was assessed according to the National Cancer Institute Common Terminology Criteria for Adverse Events (CTCAE). Tumor response was assessed using the modified Response Evaluation Criteria in Solid Tumors (mRECIST) and RECIST v1.1 (22, 23).

### Statistical Analyses

Safety and efficacy analyses were conducted for all patients who received at least one cycle of triple combination therapy and completed clinical evaluation. For baseline characteristics, variables were expressed as frequencies and percentages, and data were expressed as either median (standard deviation) or mean (range). Survival data were analyzed using the Kaplan–Meier method. OS was calculated from the date of initiation of triple combination therapy until death or last follow-up. PFS was calculated from the date of initiation of triple combination therapy until disease progression, recurrence or last follow-up. All data were analyzed using SPSS version 23.

## Results

### Patients

**Figure 1:**
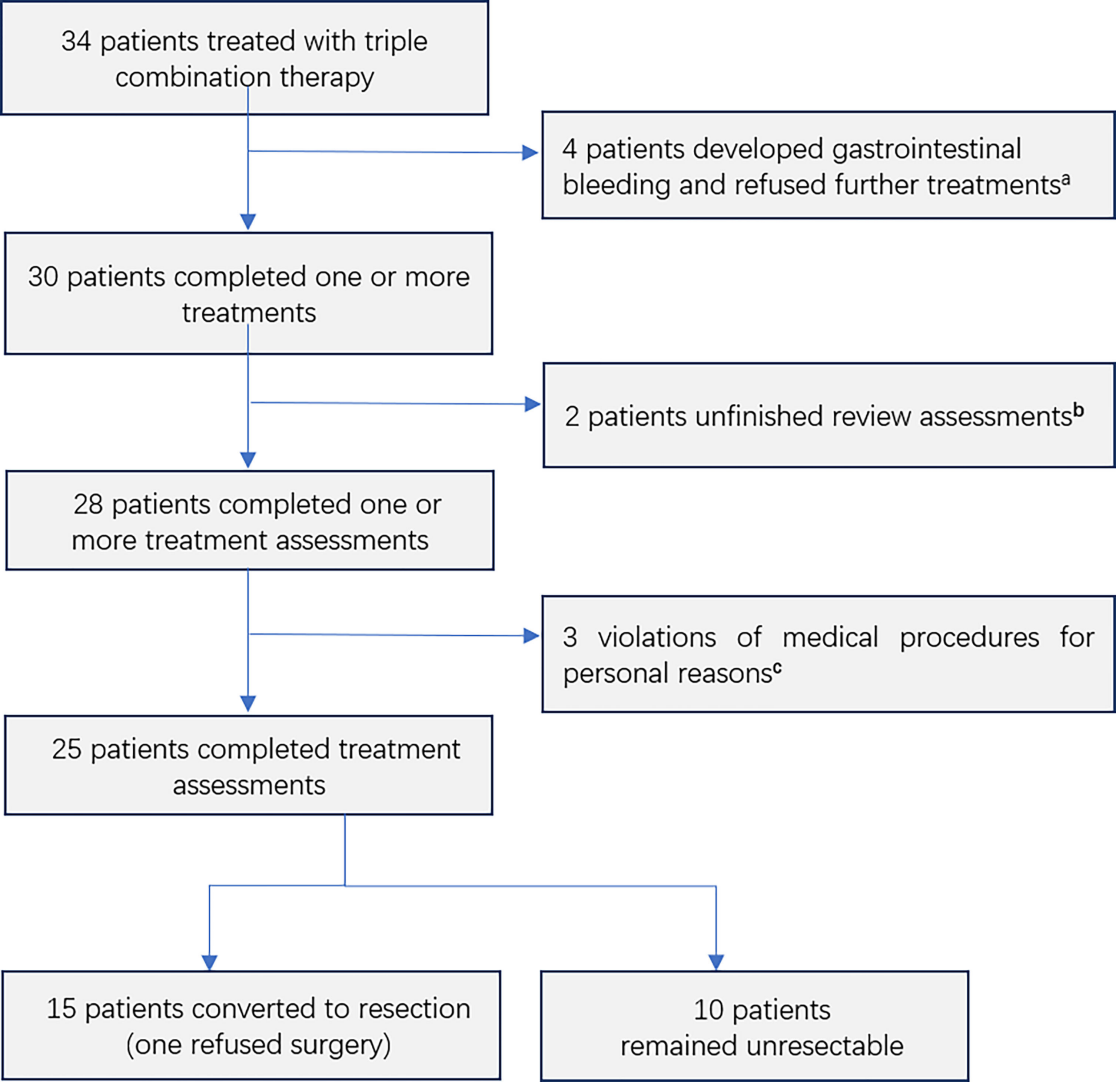
Consolidated Standards of Reporting Trials diagram including all 34 patients who entered into the study. ^a^All four patients had gastric fundus varices owing to portal hypertension. Two patients had grade 3 gastrointestinal bleeding, which was treated endoscopically. Two patients had grade 1 gastrointestinal bleeding, which was not treated. ^b^Two patients did not follow the procedure requiring a review of imaging 4–8 weeks after the last HAIC treatment. ^c^The interval between the two HAIC treatments was >8 weeks for personal reasons.

A total of 34 patients had received triple combination therapy, of whom four failed to complete the procedure because of gastrointestinal bleeding (two with grade 3 and two with grade 1), two did not complete the procedural requirement to perform imaging tests 4–8 weeks after the last HAIC treatment and three had a HAIC interval >8 weeks for personal reasons. Ultimately, 25 patients [19 males and six females; median age: 59 years (range: 49–78 years)] were included in the analysis (**Figure 1**). The follow-up ended on May 1, 2021.

All patients included in the analysis had HCC considered unresectable for three reasons: 1) the main portal vein or inferior vena cava were invaded (n = 10); 2) multiple lymph node metastases or intrahepatic lesions could not be radically resected (n = 6); 3) the residual liver volume was insufficient after radical resection (n = 9). Of these 25 patients 100% had BCLC stage C disease, 22 (88.0%) had Child–Pugh grade A liver function and three (12.0%) had Child–Pugh grade B, 22 (88.0%) had hepatitis B and two (8.0%) had hepatitis C, 20 (80.0%) had liver cirrhosis, six (24.0%) had esophago-gastric varices and 23 (92.0%) had elevated alpha-fetoprotein (AFP) levels (AFP >7 ng/mL), including 10 (40.0%) with AFP >1000 ng/mL. At baseline, according to the Japanese grading system for tumor emboli (24), there were 18 patients with portal vein invasion (Vp2, Vp3, and Vp4 grades: four, seven, and seven patients, respectively), four patients with hepatic vein invasion (Vv2 and Vv3: two and two patients, respectively), and one patient with both invasion of the portal vein and the inferior vena cava (Vp3 and Vv3, one patient). In addition, ten patients with an invasion of the main trunk of the portal vein (Vp4, n = 7) or inferior vena cava (Vv3, n = 3) were defined as “super-advanced” patients. The number of patients with multiple intrahepatic foci and extrahepatic lymph node metastases, confirmed by imaging, was six (24.0%) and 12 (48.0%), respectively (**Table 1**).

**Table 1:** Baseline demographic and clinical characteristics (n = 25).

Characteristics	No.
Age, years	
Median	61.95
Range	49–78
Gender	
Male	19
Female	6
Hepatitis B virus infection	22
Hepatitis C virus infection	2
Child-Pugh classification	
A	22
B	3
BCLC stage	
B	0
C	25
Serum AFP level, ng/mL	
<400	11
400–1,000	4
≥1,000	10
Liver cirrhosis	20
Esophago-gastric varices	6
Macroscopic vascular invasion^a^	
Vp2	4
Vp3	7
Vp4	7
Vv2	2
Vv3	2
Vp3 & Vv3	1
Lymphatic metastasis	12
Intrahepatic metastasis	6

a Vp2, invasion of (or tumor thrombus in) second order branches of the portal vein; Vp3, invasion of (or tumor thrombus in) first order branches of the portal vein; Vp4, invasion of (or tumor thrombus in) the main trunk of the portal vein and/or contra-lateral portal vein branch to the primarily involved lobe; Vv2, invasion of (or tumor thrombus in) the right, middle, or left hepatic vein, the inferior right hepatic vein, or the short hepatic vein; Vv3, invasion of (or tumor thrombus in) the inferior vena cava (24).

BCLC, Barcelona Clinic Liver Cancer; PVTT, portal vein tumor thrombus; IVCTT, inferior vena cava tumor thrombus.

### Treatment

For angiogenesis inhibitor, patients chose apatinib (six patients, 125–250 mg/day, mean 225 mg/day), lenvatinib (18 patients, 4–8 mg/day, mean 6.87 mg/day), or sorafenib (one patient, 400 mg BID), based mainly on their economic situation. All patients also received anti-PD-1 monoclonal antibodies intravenously every 3 weeks (13 patients received camrelizumab and 12 sintilimab). In general, five different drug regimens were used, including sorafenib with camrelizumab (n = 1), apatinib with camrelizumab (n = 5), lenvatinib with camrelizumab (n = 7), apatinib with sintilimab (n = 2) and lenvatinib with sintilimab (n = 10). The mean HAIC interval was 45.9 days, the mean number of doses received was 2.96 (patients who achieved conversion to surgical treatment received fewer courses of HAIC than the non-converted group: 2.47 *vs.* 4.13) and the mean dose of the component therapies received was: oxaliplatin, 72.9 mg/m^2^; calcium folinate, 269.2 mg/m^2^; fluorouracil bolus, 341.2 mg/m^2^; and fluorouracil infusion, 971.4 mg/m^2^.

### Safety

Most patients (92.0%, 23/25) experienced adverse events with varying severity (**Table 2** and **Supplementary Table 1**), with 84.0%, 68.0%, 28.0%, and 0% of patients experiencing grade 1, 2, 3 and 4-related adverse events, respectively. The most common treatment-related adverse events of any grade were neutropenia (36.0%, n = 9), leukopenia (32.0%, n = 8), elevated aspartate aminotransferase levels (28.0%, n = 7), anemia (28.0%, n = 7), elevated alanine aminotransferase levels (24.0%, n = 6), and hypoproteinemia (24.0%, n = 6). Treatment-related adverse events resulted in three lenvatinib treatment dose reductions (17.6% of all lenvatinib patients) and one apatinib treatment dose reduction (14.2% of all apatinib patients). Treatment-related adverse events resulted in reduced doses of HAIC in 10 (40.0%) patients. Two (8.0%) patients received endoscopic hemostasis for gastrointestinal bleeding (grade 3) that delayed HAIC treatment.

**Table 2:** Summary of the most common treatment-related adverse events in patients with advanced hepatocellular carcinoma receiving triple therapy (n = 25).

Preferred AE term, n (%)	Any grade	Grade 1	Grade 2	Grade 3
Neutropenia	9 (36.0)	3 (12.0)	6 (24.0)	0
Leukopenia	8 (32.0)	2 (8.0)	5 (20.0)	1 (4.0)
Anemia	7 (28.0)	4 (16.0)	3 (12.0)	0
AST level increased	7 (28.0)	2 (8.0)	3 (12.0)	2 (8.0)
ALT level increased	6 (24.0)	3 (12.0)	2 (8.0)	1 (4.0)
Hypoalbuminemia	6 (24.0)	6 (24.0)	0	0
Serum bilirubin increase	5 (20.0)	4 (16.0)	1 (4.0)	0
Rash	5 (20.0)	2 (8.0)	3 (12.0)	0
Hypertension	4 (16.0)	0	3 (12.0)	1 (4.0)
Hyperglycemia	4 (16.0)	4 (16.0)	0	0
Oulorrhagia	4 (16.0)	3 (12.0)	1 (4.0)	0
Fatigue	3 (12.0)	3 (12.0)	0	0
Proteinuria	3 (12.0)	0	3 (12.0)	0
Diarrhea	2 (8.0)	2 (8.0)	0	0
Nausea	2 (8.0)	2 (8.0)	0	0
Pruritus	2 (8.0)	1 (4.0)	1 (4.0)	0
Edema peripheral	2 (8.0)	2 (8.0)	0	0
Epistaxis	2 (8.0)	2 (8.0)	0	0
Decreased appetite	2 (8.0)	2 (8.0)	0	0
Gastrointestinal bleeding	2 (8.0)	0	0	2 (8.0)
Hypothyroidism	1 (4.0)	0	1 (4.0)	0
Weight decreased	1 (4.0)	1 (4.0)	0	0
Abdominal distention	1 (4.0)	1 (4.0)	0	0
Arthralgia	1 (4.0)	1 (4.0)	0	0
Gastrohelcoma	1 (4.0)	0	1 (4.0)	0

AE, adverse event; ALT, alanine aminotransferase; AST, aspartate transaminase.

### Tumor Response

Treatment efficacy was evaluated based on the investigator’s assessment using mRECIST and RECIST v1.1. However, for consistency, only assessments using mRECIST are summarized in the following section. The best ORR was 96.0% and the median TTR was 50.5 days (95% CI: 31.02–64.00) (**Table 3**). A CR was observed in 12 patients (48.0%), a PR in 12 patients (48.0%), and stable disease (SD) in one patient (4.0%). The efficacy of the different drug combinations was summarized and all showed satisfactory results (**Supplementary Table 3**). During the follow-up period, three patients progressed from a PR to PD and eventually died. One of them progressed during treatment and had a time-to-progression (TTP) of 182 days. The other two discontinued interventional treatment after two cycles of HAIC owing to the COVID-19 pandemic.

**Table 3:** Summary of efficacy outcomes (n = 25).

Variables, n (%)	Best overall response (mRECIST)	Best overall response (RECIST v1.1)	Overall response at data cut-off (mRECIST)	Beyond criteria^a^ best response (mRECIST)	Under criteria^b^ best response (mRECIST)
Complete response	12 (48.0)	2 (8.0)	12 (48.0)	4 (40)	8 (53.3)
Partial response	12 (48.0)	19 (76.0)	9 (36.0)	6 (60)	6 (40.0)
Stable disease	1 (4.0)	4 (16.0)	1 (4.0)	0 (0)	1 (6.7)
Progressive disease	0 (0)	0 (0)	3 (12.0)	0 (0)	0 (0)
Objective response rate	24 (96.0)	21 (84.0)	21 (84.0)	10 (100)	14 (93.3)
Received hepatic resection	14 (56.0)	14 (56.0)	14 (56.0)	5 (50)	9 (60.0)
Pathologic complete response	7 (28.0)	7 (28.0)	7 (28.0)	2 (20)	5 (33.3)

a Beyond criteria included patients with vein tumor thrombus Vp4 or Vv3;

b Under criteria included patients without the above states.

Among the nine patients who could not complete treatment, seven had an imaging review; two of these achieved PR, one had PD and four had SD. The last two patients had no imaging review and could not be evaluated for efficacy (see **Supplementary Table 2** for all 32 patients who were treated and had imaging evaluations).

### AFP Response to Treatment

Before treatment, the median AFP level was 539.30 ng/mL (95% CI: 82.82–1310.00) and decreased to 10.20 ng/mL (95% CI: 4.64–28.32) after the first cycle of treatment. As of March 1, 2021, in 19 patients (82.6% of patients with elevated AFP at baseline) AFP levels had returned to the normal range (<7 ng/mL). The AFP level changes in all 25 patients are shown in **Figure 3E**.

### Long-Term Outcomes

Fifteen patients (60.0%) met the surgical criteria for tumor and embolus regression; 14 (56.0%; one refused surgery) underwent surgical resection; and seven (28.0%) achieved a pathologic CR that was confirmed post-operatively. One patient had post-operative intrahepatic recurrence, whose relapse-free survival (RFS) was 13.17 months.

**Figure 2:**
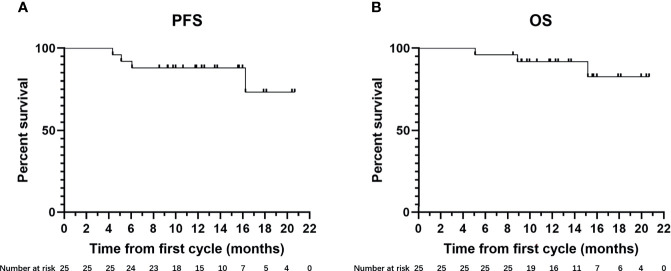
Kaplan-Meier estimates of **(A)** progression-free survival (PFS) by modified RECIST (n = 25) and **(B)** overall survival (OS) (n = 25).

**Figure 3:**
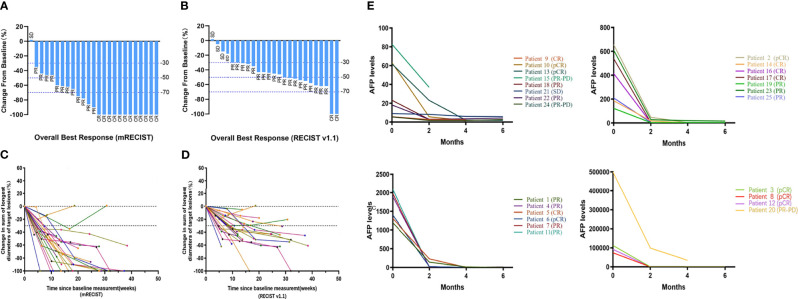
Best percentage change in tumor burden and changes of AFP levels over time. Waterfall plot of best percentage change from baseline in sums of diameters of target lesions by **(A)** modified RECIST and **(B)** RECIST v1.1; Percentage change in target lesions diameters over time by **(C)** modified RECIST and **(D)** RECIST v1.1; **(E)** AFP levels of 25 patients before treatment, two months, four months and six months after first cycle of treatment, presented with their efficacy outcomes by modified RECIST, respectively.

Of the 25 patients included in the analysis, 14 (56%) were followed up for more than 12 months (median follow-up time: 15.85 months [range: 12.30–20.67 months]), and their 6-month and 12-month PFS rates were 92.9% and 92.9%, respectively. The other 11 patients (44%) were followed up for less than 12 months [median follow-up time: 9.73 months (range: 5.10–11.83 months)] had a 6-month PFS rate of 72.70%. For all patients in the analysis, the median follow-up duration was 12.53 months (interquartile range, 9.85–16.95 months) and during this time the median PFS and median OS was not reached (**Figure 2**). The 6-month PFS rate for the ten non-converted patients was 70%. Among the seven patients who survived but did not achieve surgical conversion, three had tumor volume reduction but not sufficiently to undergo radical surgery, three had tumor volume reduction but lymph node metastasis did not completely disappear, and one patient had no obvious change in tumor volume. The changes in maximum tumor diameter after treatment are presented in **Figure 3**, and representative imaging data are presented in **Supplementary Figures 1** and **2**.

## Discussion

Various combinations of treatments have been investigated for achieving surgical conversion in patients with advanced HCC including transarterial chemoembolization, HAIC, immuno-therapy, chemoradiotherapy, and systemic chemotherapy. However, the reported conversion rates are far from satisfactory at 15-20% (25). Recent studies have suggested that the combination of HAIC-based locoregional therapy with targeted therapy and immunotherapy is a promising multimodal approach for advanced HCC (20). In the present study, we demonstrated a high ORR (96.0%) achieved in a relatively short time (median TTR 50.5 days) compared with previous reports of combination regimens (ORR: 23.1–54.4%) with the triple combination of angiogenesis inhibitors, PD-1 inhibitors and HAIC with FOLFOX, as well as a relatively high surgical conversion rate (60.0%) (3, 6–9, 11, 14). Our study investigated the combined effects of drugs with different mechanisms without limiting the types or brands of angiogenesis inhibitors and PD-1inhibitors. However, all combination regimens investigated in this study showed similar positive results, indicating universal applicability of the triple combination therapy. Furthermore, the high pathologic CR rate suggests that triple combination therapy can provide greater survival benefits, which is supported by the favorable 6- and 12-month PFS. However, evaluation of median overall survival requires an extended follow-up period and could not be evaluated in this analysis.

Ninety-two percent of the patients in this analysis had macrovascular invasion (n = 23), including 10 patients (40.0%) with invasion of the main trunk of the portal vein or inferior vena cava, which was an exclusion criteria in most previous clinical trials of treatments for HCC. Although the small sample size of this study may have led to biased results, the results showed that this group of “super-advanced” patients had an ORR of 100% following triple therapy and a surgical conversion rate of 50.0% (5/10), including four patients (40.0%) who achieved a pathologic CR (**Table 3**). This result needs further validation over a more extended follow-up period.

**Figure 4:**
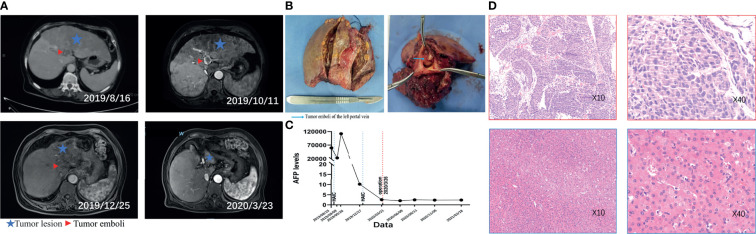
Representative MRI images, AFP levels and pathological findings highlighting changes before and after treatment in one patient. The patient was diagnosed with unresectable HCC in August 2019 and began triple combination therapy. First HAIC was performed on August 27, 2019, and the patient had endoscopic haemostasis for gastrointestinal bleeding (grade 3) that delayed the subsequent HAIC treatment. A second round of HAIC was performed on December 31, 2019, and the patients underwent surgery on March 26, 2020. Preoperative imaging evaluation showed a partial response, and postoperative pathology showed a pathologic complete response. **(A)** Imaging changes before and after treatment (comparison of tumor size at different times was marked by the sagittal segment of the portal vein. Aftertreatment, the volume of the left lobe of the liver was significantly reduced, and the right lobe was compensated); **(B)** intraoperative gross pathological specimen (left), transverse section of left portal vein (right); **(C)** change of AFP levels over time; **(D)** preoperative diagnostic pathological picture (above), postoperative pathological picture (below).

The triple therapy protocol described in this study rapidly reduced tumor load and attenuated tumor activity, manifested by a rapid decrease in AFP level and tumor volume (**Figure 3**), extensive tumor necrosis and inactivation of cancer thrombus regression (**Supplementary Figures 1, 2**). These manifestations can be observed in data from a typical patient who had esophago-gastric varices and then grade 3 gastrointestinal bleeding after first cycle of treatment. Although the endoscopic hemostasis delayed subsequent treatment and AFP level was elevated, the tumor and emboli of the left portal vein showed complete necrosis after the second cycle of triple combination therapy (**Figure 4**). This high level of anti-tumor activity led to 60% of patients included in the analysis, all of whom had initially unresectable tumors, achieving sufficient reductions in tumor load to meet the standard for radical resection. Every one of the 15 converted patients received no more than three courses of HAIC treatment (mean 2.37 courses), while the non-converted patients received more courses (mean 4.13 courses), suggesting that rapid tumor response to treatment is associated with a high rate of conversion.

Surgical resection allowed patients to minimize tumor load before tumor progression due to drug resistance, thus increasing their chances of an effective cure and prolonged survival. Of the 14 patients who underwent surgery, nine (64.3%) had Vp3–4 portal vein tumor thrombosis (PVTT); after a median follow-up time of 18.13 months (range: 9.23–20.67months), only one post-operative intrahepatic recurrence was observed and treated with radiofrequency ablation. This is significant when compared with previous reports which have shown a median survival time of only 0.5–0.8 years in HCC patients with Vp3–4 PVTT (26). Furthermore, of the 14 patients who underwent resection, only the one patient who relapsed had an AFP level of >7 ng/mL after surgery, which suggests that AFP can be used as an indicator of postoperative recurrence. At the end of follow-up, the other 13 post-operative patients had no recurrence or metastasis on imaging assessment; however, median relapse-free survival could not be assessed.

The cohort of patients in this study had many characteristics usually associated with poor prognosis and limited treatment options, including a background of hepatitis [hepatitis B, n = 22 (88.0%); hepatitis C, n = 2 (8.0%)], vascular invasion [n = 23 (92.0%)], a baseline tumor diameter >10 cm [n = 13 (52.0%)], cirrhosis [n = 17 (68.0%)] and portal hypertension [n = 13 (52.0%)]. The liver function of the patients was also mostly in the compensatory stage affected by both cirrhosis and the tumor and was susceptible to deterioration by therapeutic agents. Therefore, in order to ensure safety and completion of treatment, we applied reduced doses of the therapeutic drugs, appropriately prolonged the treatment period and maintained continuous treatment for patients with poor liver function and poor tolerance of side effects. As the triple combination regimen rapidly reduced the tumor load and retracted the vascular carcinoma thrombus, the liver blood supply improved, increasing tolerance to the treatment. As a result, no grade 4 adverse events occurred and only two patients with portal hypertension experienced gastrointestinal bleeding that resulted in treatment delay.

The reasons for failure to complete treatment according to the protocol were: 1) the occurrence of the COVID-19 pandemic during the treatment period, which made it difficult for patients to seek medical attention, especially trans-provincial patients who could not be treated and re-examined on time; 2) fear of complications of treatment; and 3) financial reasons. Presently, the COVID-19 outbreak has been effectively controlled in China, and the difficulties of patients seeking treatment have been resolved. Regarding complications, the triple combination therapy demonstrated a high level of safety with no grade 4 treatment-related side effects or treatment-related deaths throughout the analysis period. Treatment-related side effects were manageable with symptomatic treatment and adjustment of the drug dosage. For example, gastrointestinal bleeding was safely managed with endoscopic therapy. The inactivation and regression of the main stem thrombus due to the treatment can also reduce the portal pressure and risk of rebleeding. Moreover, for patients with severe gastric fundus varices, endoscopic ligation before treatment can also reduce the risk of severe bleeding. Financially, the triple combination protocol allows for various combinations of different angiogenesis inhibitors and PD-1 inhibitors, which provides patients with options that suit their economic circumstances. Furthermore, our study extended the treatment interval of HAIC to 4–8 weeks, reducing the number of hospitalizations and medical costs, with the efficacy of treatment unaffected.

This study has several limitations. Firstly, this was a single-center, retrospective, single-arm study with a small sample size which is likely to have led to selection bias and does not provide a comparator for the experimental therapy. Prospective studies with a larger population from multiple centers are needed to verify the results. Secondly, patients in this study received different combinations of angiogenesis inhibitors and PD-1 inhibitors. The preliminary conclusion that the different drug combinations were all beneficial warrants further validation in a larger sample. Finally, the baseline characteristics of patients were different for the subgroups with regards to macrovascular invasion, tumor burden, and non-tumor liver histology, and this may have affected the clinical outcome and side effects of treatment.

In conclusion, a triple combination therapy comprised of angiogenesis inhibitors, PD-1inhibitors and HAIC with FOLFOX had a significant therapeutic effect in patients with initially unresectable locally advanced HCC and was associated with an extremely high surgical conversion rate. Toxic effects were manageable, and our findings suggest there will be long-term efficacy.

## Data Availability Statement

The raw data supporting the conclusions of this article will be made available by the authors, without undue reservation.

## Ethics Statement

The studies involving human participants were reviewed and approved by The Research Ethics Committee of Tianjin Medical University Cancer Institute and Hospital. The patients/participants provided their written informed consent to participate in this study.

## Author Contributions

TZ: study concept and design. JZ and XZ: acquisition and analysis or interpretation of data. JZ: drafting of the manuscript. TZ and LW: critical revision of the manuscript. JZ and XZ: statistical analysis. WX, HM, and GY: administrative and technical support. All authors contributed to the article and approved the submitted version.

## Funding

This study was funded by the National Science and Technology Major Project (No. 2017ZX10203207) and by the National Natural Science Foundation of China (No. 81672884). The National Science and Technology Major Project and the National Natural Science Foundation of China had no role in the design or conduct of the study; collection, management, analysis, and interpretation of the data; preparation, review, or approval of the manuscript; or the decision to submit for publication.

## Conflict of Interest

The authors declare that the research was conducted in the absence of any commercial or financial relationships that could be construed as a potential conflict of interest.

The reviewer HS declared a shared parent affiliation with one of the authors LW, to the handling editor at time of review.

## Publisher’s Note

All claims expressed in this article are solely those of the authors and do not necessarily represent those of their affiliated organizations, or those of the publisher, the editors and the reviewers. Any product that may be evaluated in this article, or claim that may be made by its manufacturer, is not guaranteed or endorsed by the publisher.
