# Supplementary material for: Surgical Conversion for Initially Unresectable Locally Advanced Hepatocellular Carcinoma Using a Triple Combination of Angiogenesis Inhibitors, Anti-PD-1 Antibodies, and Hepatic Arterial Infusion Chemotherapy: A Retrospective Study
Source: Front Oncol. 2021 Nov 12;11:729764. doi: 10.3389/fonc.2021.729764 (PMC8632765; doi:10.3389/fonc.2021.729764)
Supplement: Supplementary file 1 [file DataSheet_1.zip › Supplementary Figure 3.DOCX]

**S-****Figure 3.** Changes of alpha-fetoprotein levels in patients before and after treatment (n = 25).


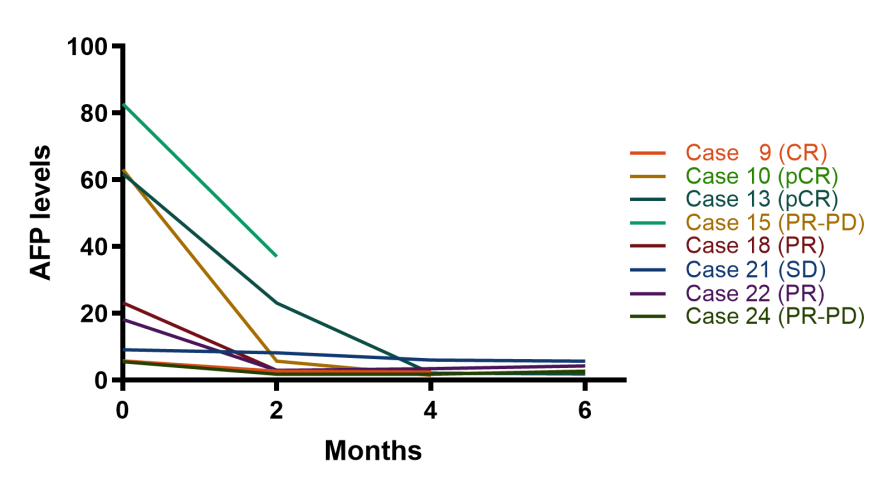


A


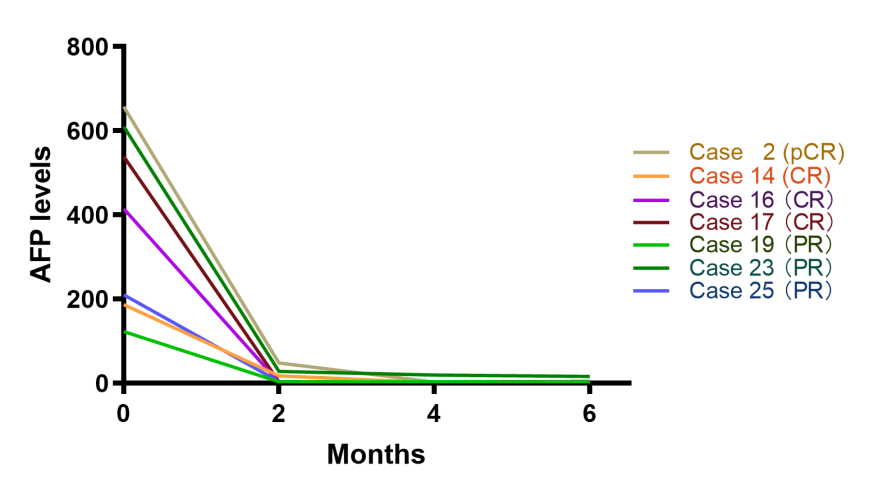


B


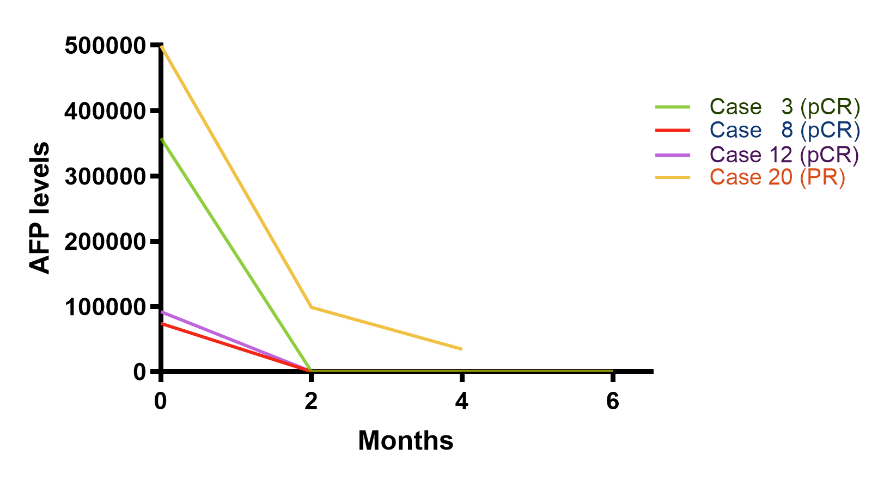


D


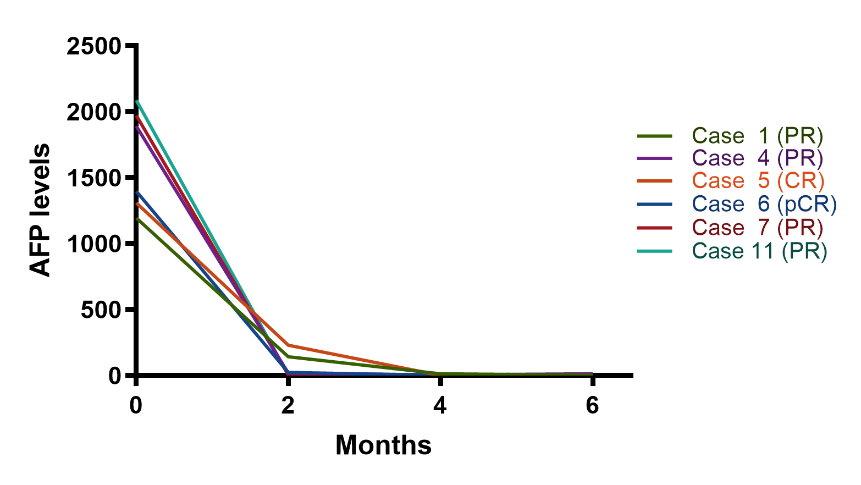


C

AFP levels of 25 patients before treatment, two months, four months and six months after first cycle of treatment, presented with their efficacy outcomes by modified RECIST, respectively. A: patient 9, 10, 13, 15, 18, 21, 22 and 24; B: patient 2, 14, 16, 17, 19, 23 and 25; C: patient 1, 4, 5, 6, 7 and 11; D: patient 3, 8, 12 and 20.

AFP, alpha-fetoprotein; CR, complete response; pCR, pathologic CR; PR, partial response; SD, stable disease; PD, progressive disease; PR-PD, disease developed from partial response to progressive disease.
