# Supplementary material for: Surgical Conversion for Initially Unresectable Locally Advanced Hepatocellular Carcinoma Using a Triple Combination of Angiogenesis Inhibitors, Anti-PD-1 Antibodies, and Hepatic Arterial Infusion Chemotherapy: A Retrospective Study
Source: Front Oncol. 2021 Nov 12;11:729764. doi: 10.3389/fonc.2021.729764 (PMC8632765; doi:10.3389/fonc.2021.729764)
Supplement: Supplementary file 1 [file DataSheet_1.zip › Supplementary Figure 4.DOCX]

**S****-****Figure 4.** Kaplan-Meier estimates of (A) progression-free survival (PFS) by modified RECIST (n = 25), (B) overall survival (OS) (n = 25).


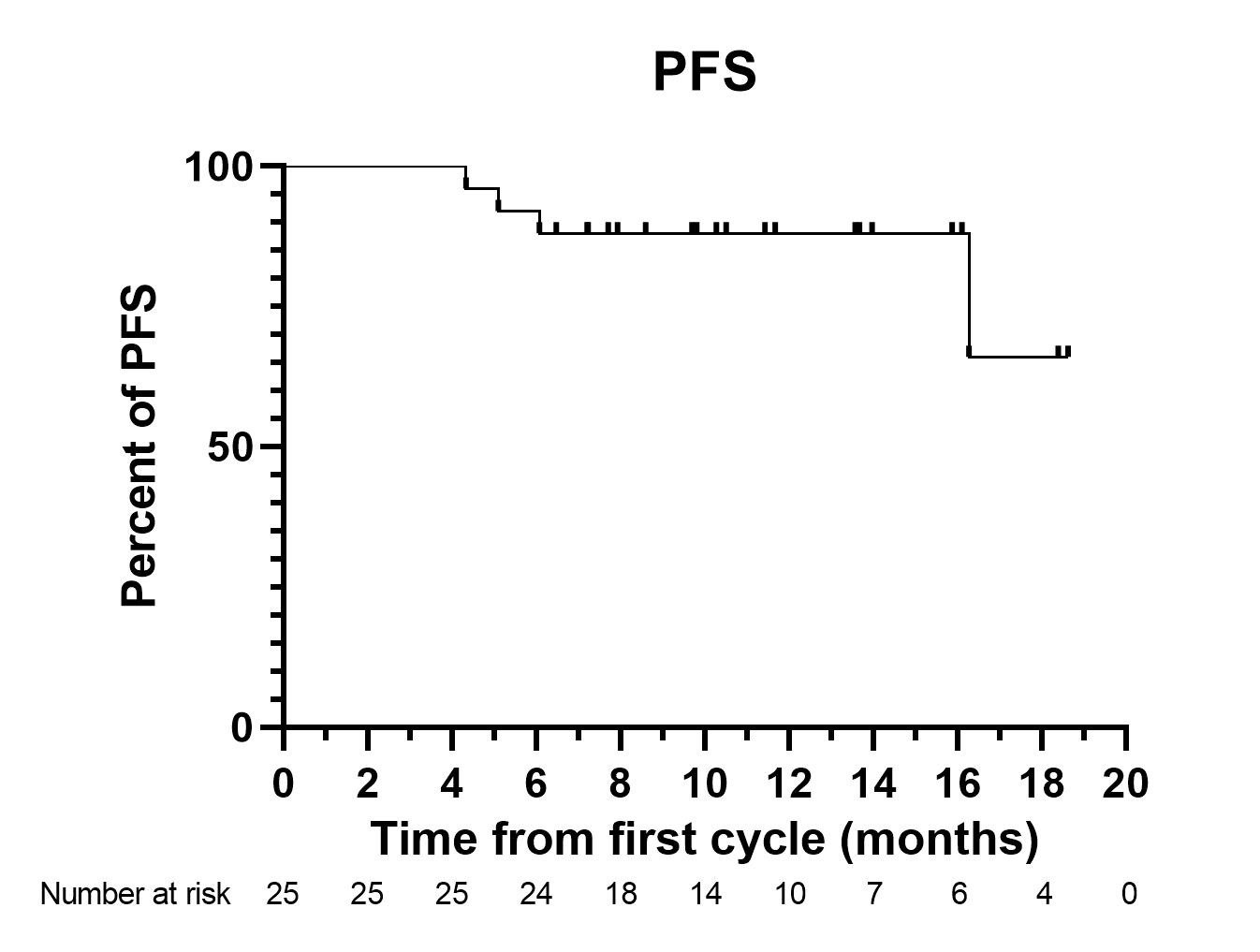


A


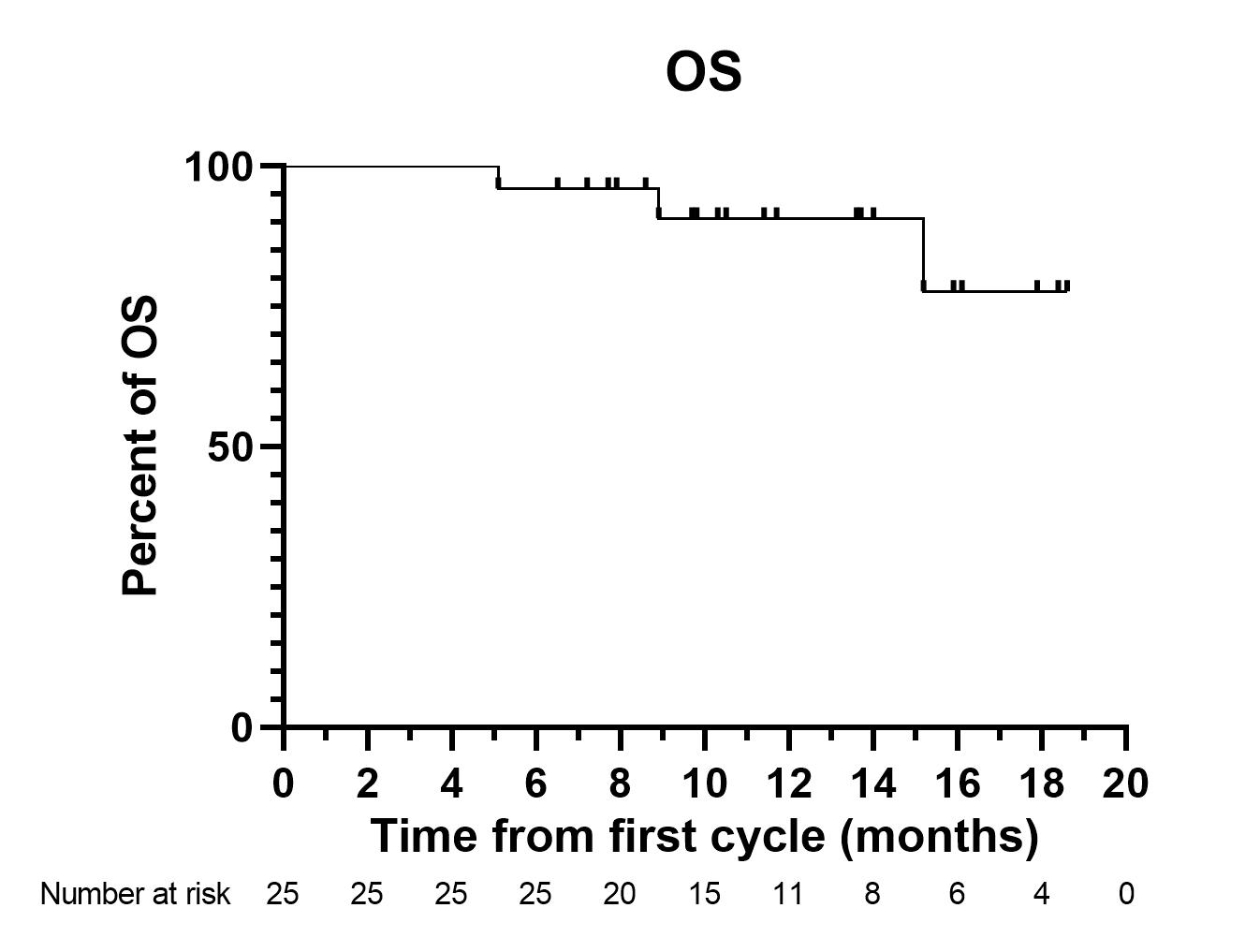


B
