# Supplementary figures and images for: Surgical Conversion for Initially Unresectable Locally Advanced Hepatocellular Carcinoma Using a Triple Combination of Angiogenesis Inhibitors, Anti-PD-1 Antibodies, and Hepatic Arterial Infusion Chemotherapy: A Retrospective Study
Source: Front Oncol. 2021 Nov 12;11:729764. doi: 10.3389/fonc.2021.729764 (PMC8632765; doi:10.3389/fonc.2021.729764)

**S-Figure 1.** Imaging manifestations of 14 surgical patients before and after treatment.

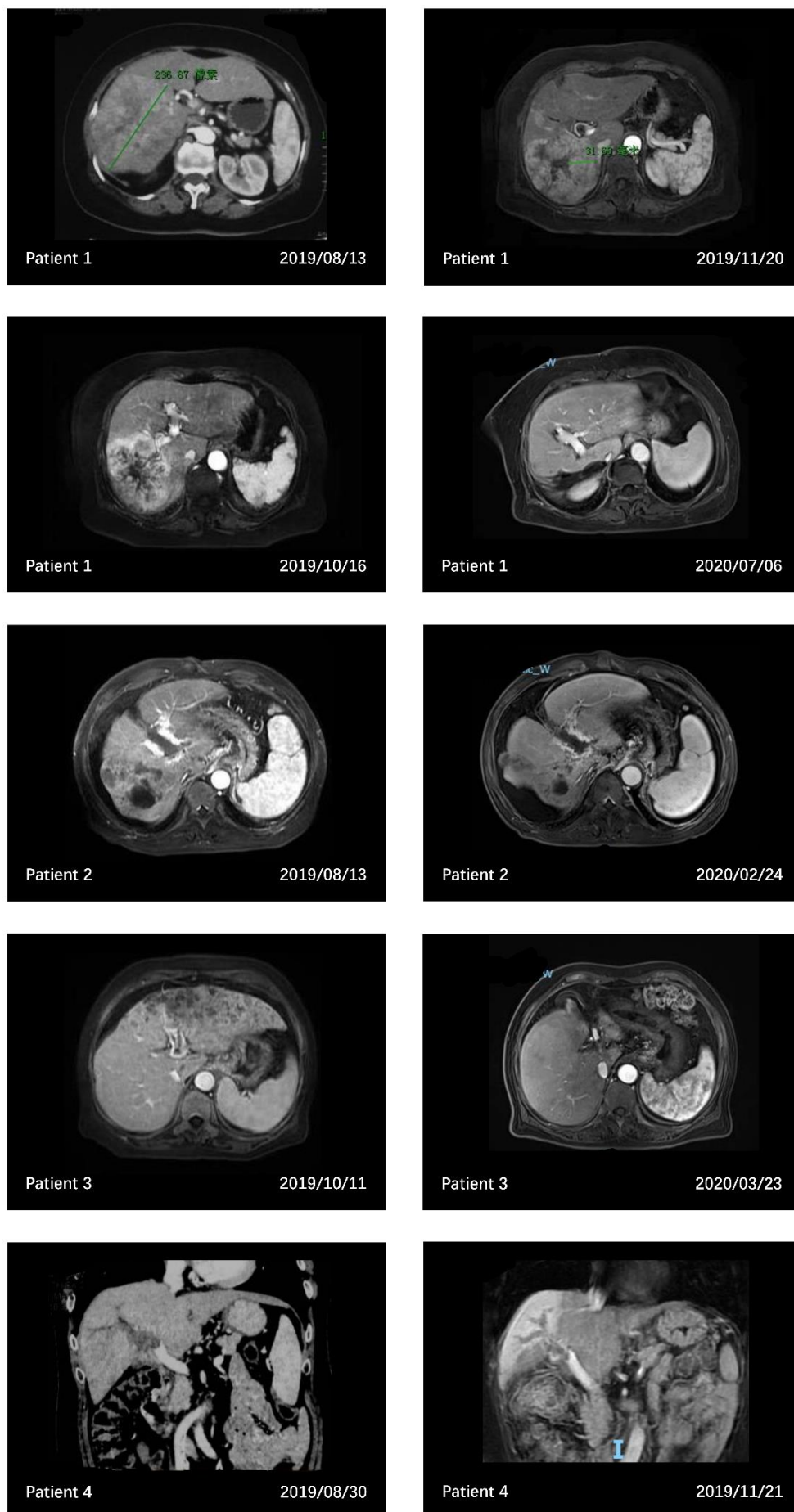

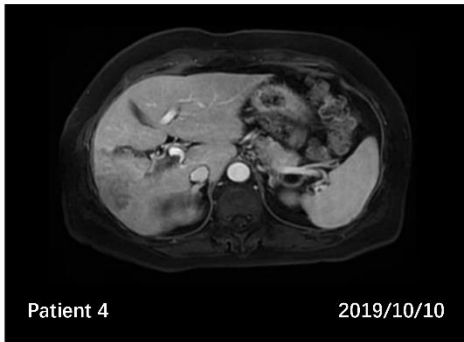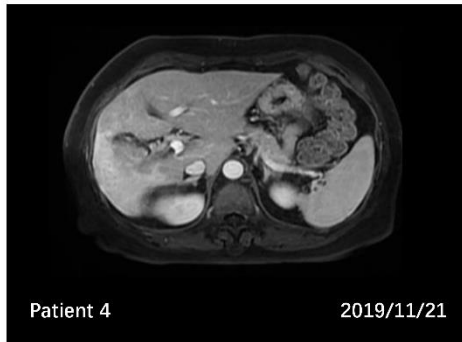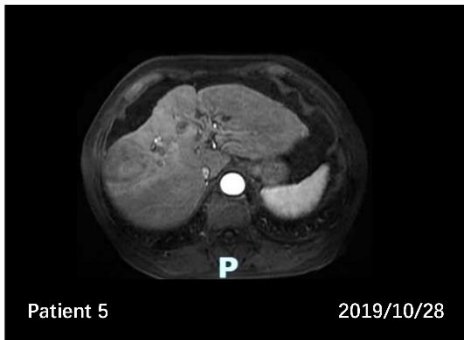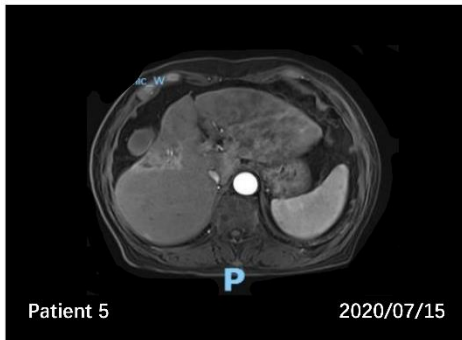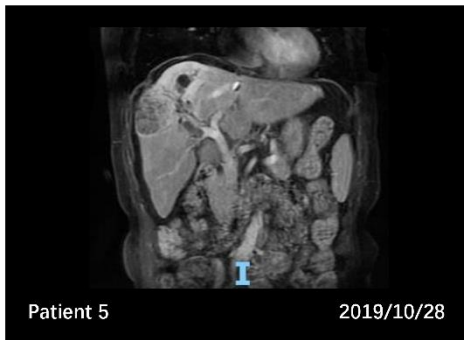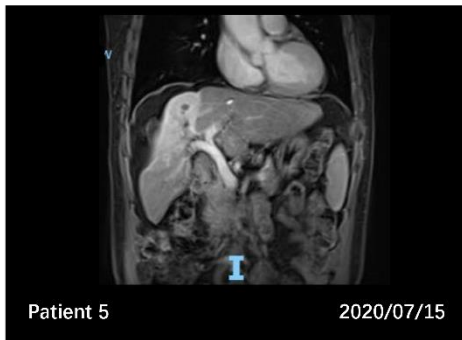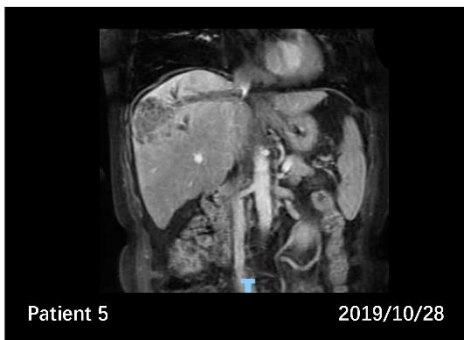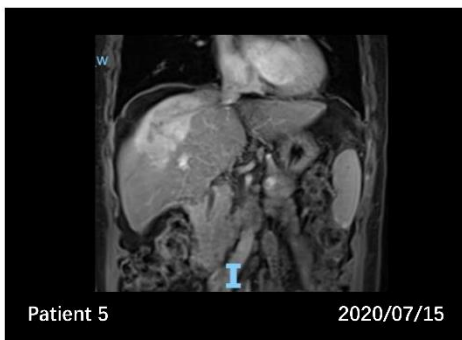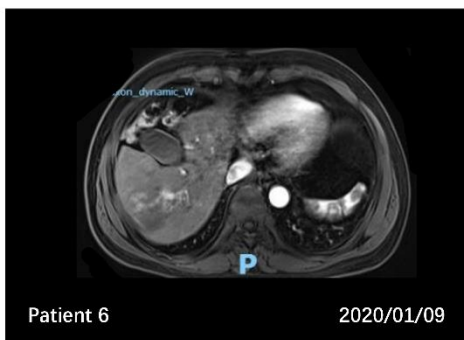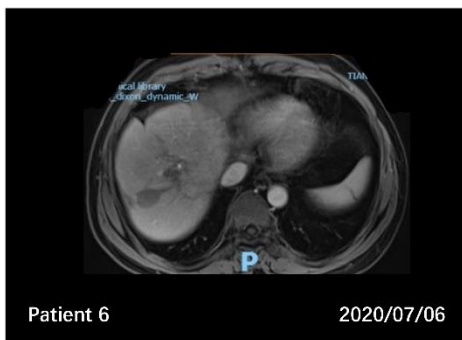

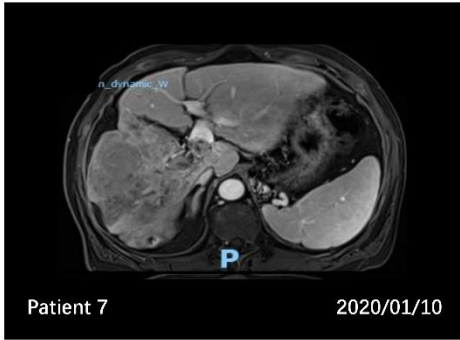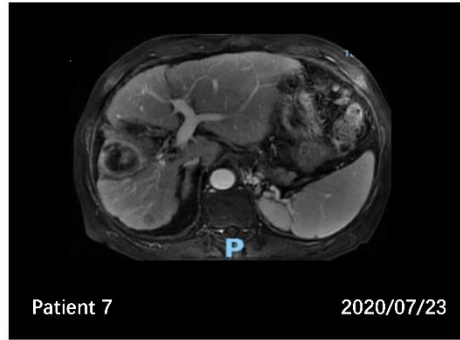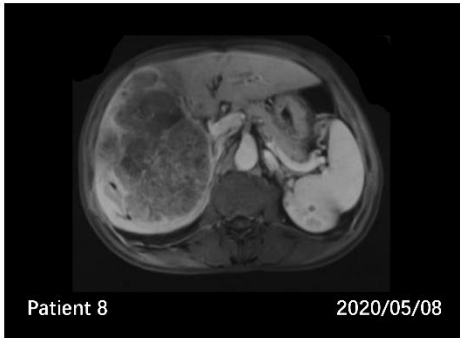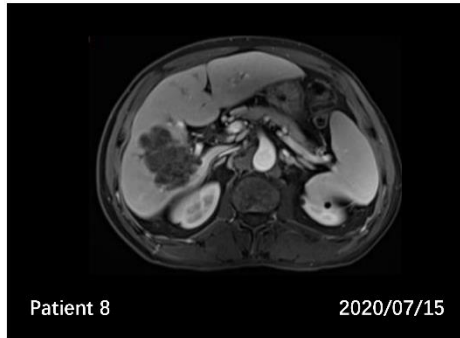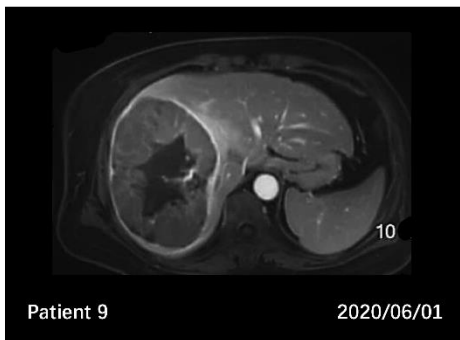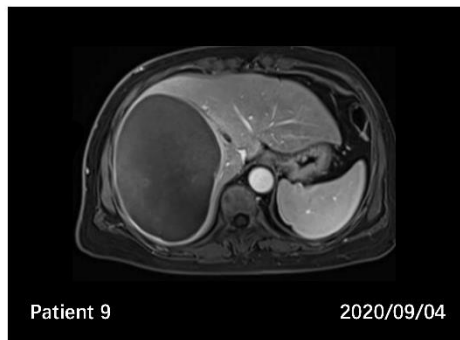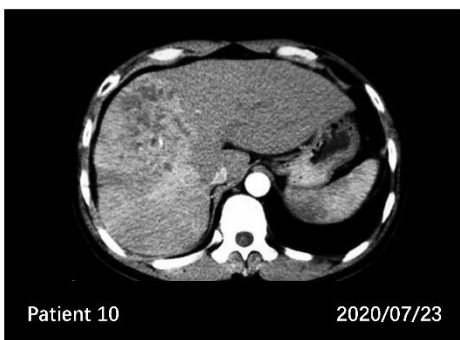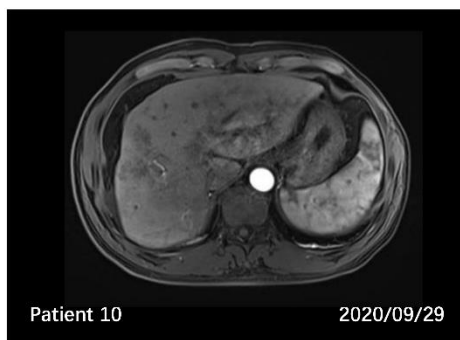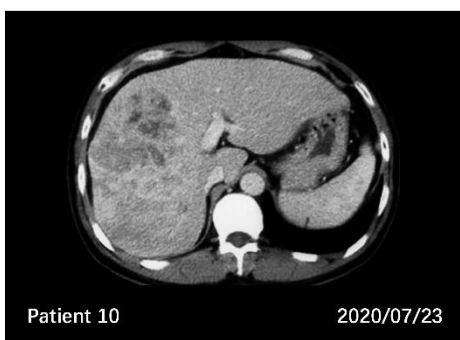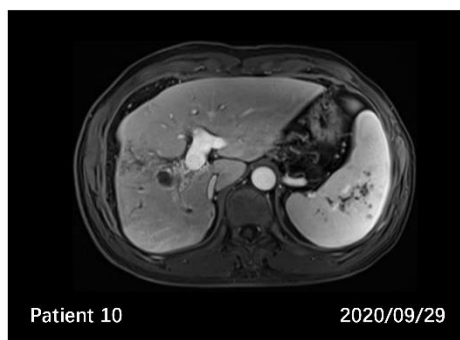

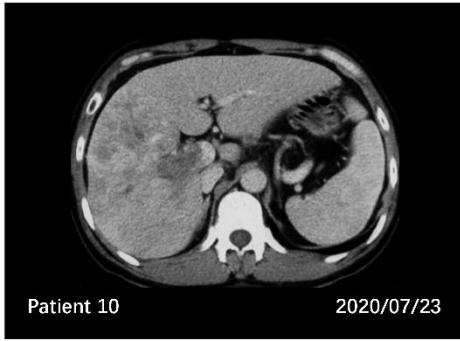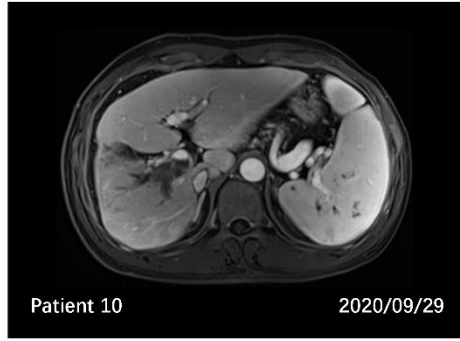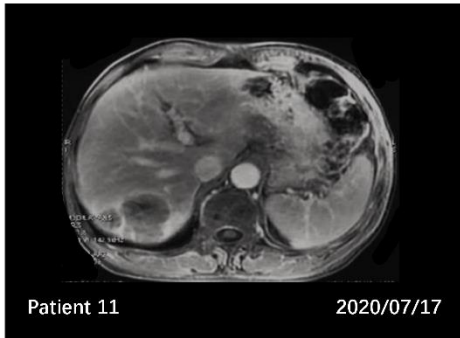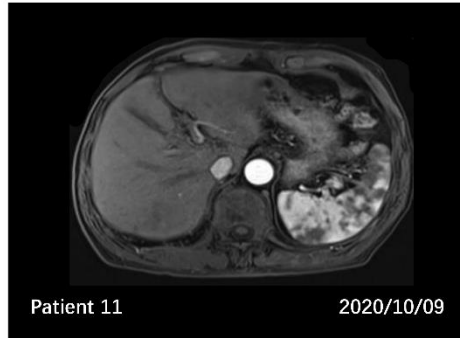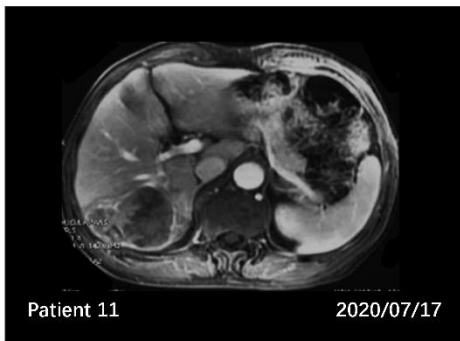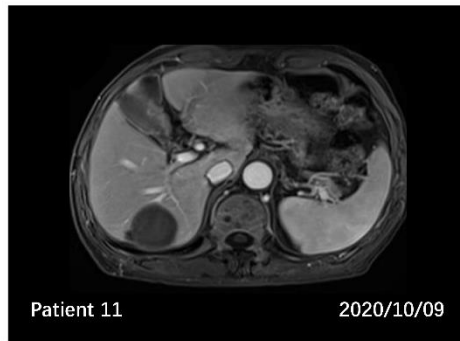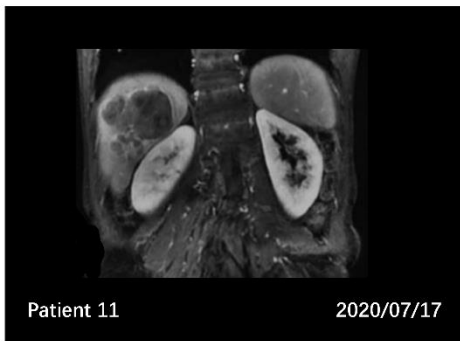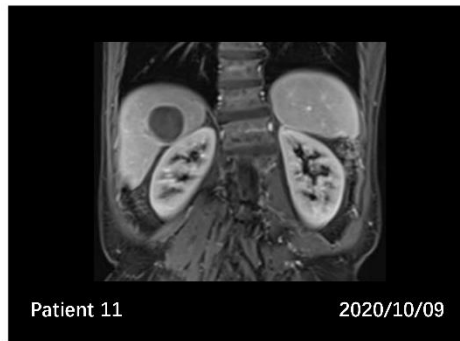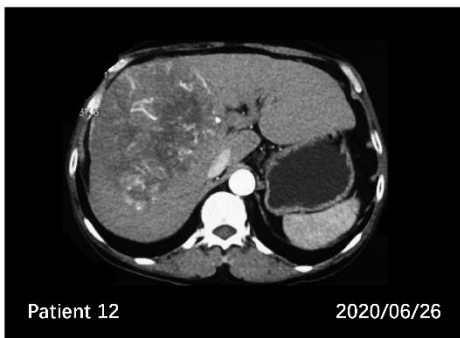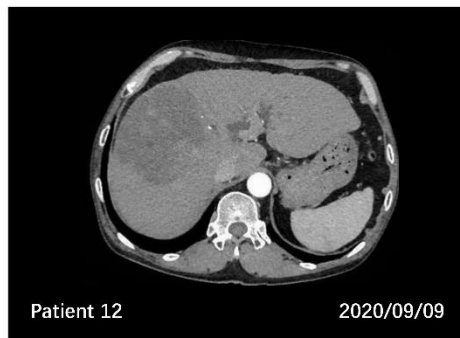

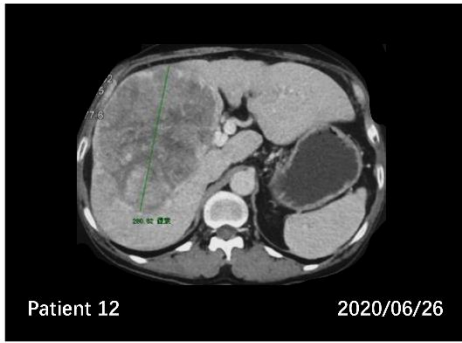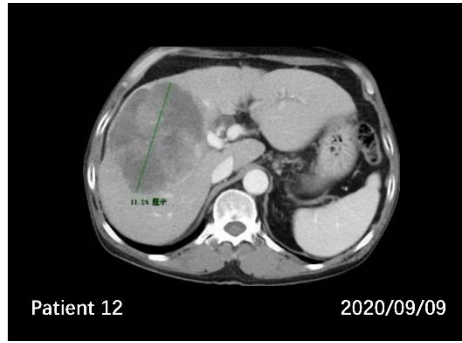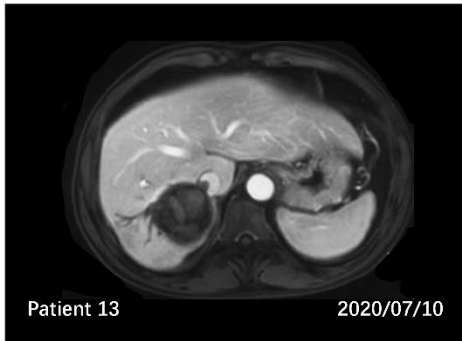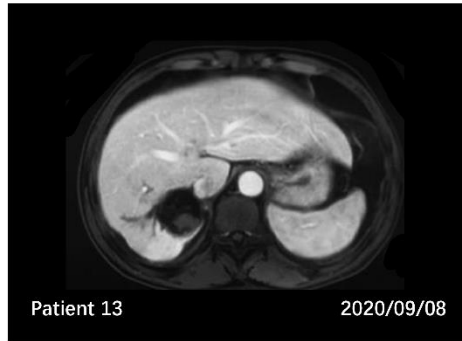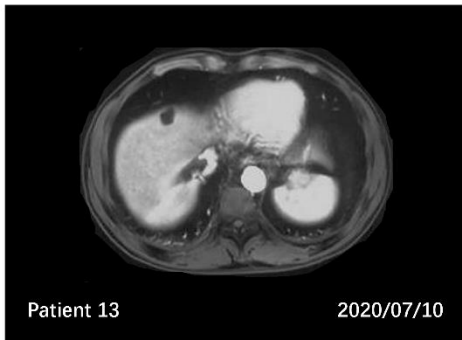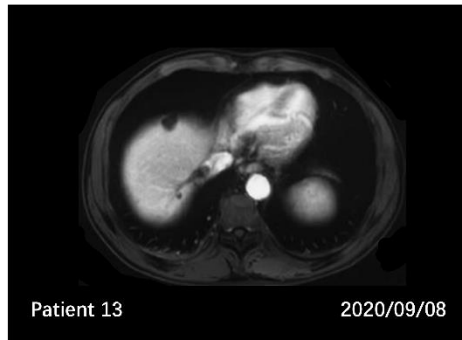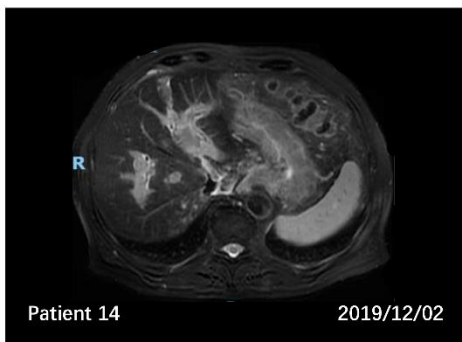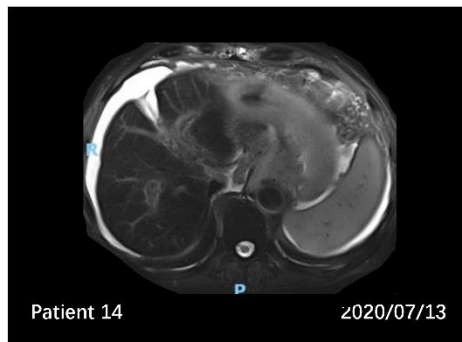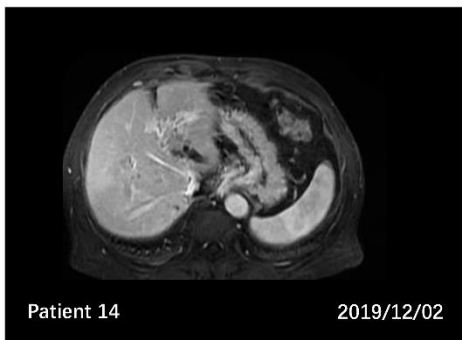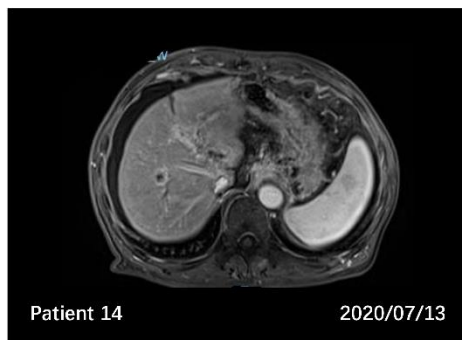

Supplement: Supplementary file 1 [file DataSheet_1.zip › Supplementary Figure 1.PDF]

**S-Figure 2.** Imaging manifestations of 11 non-surgical patients before and after treatment.

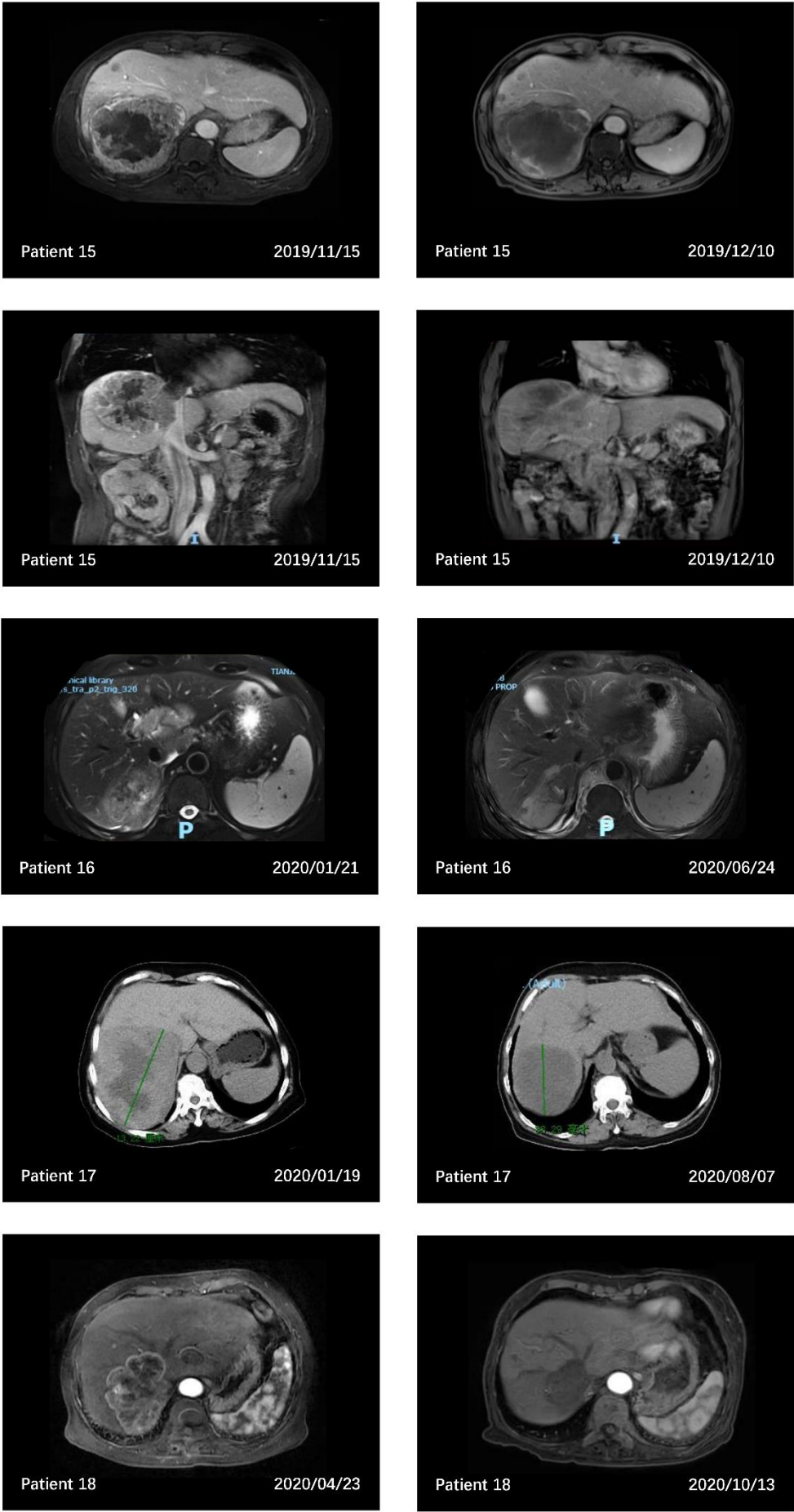

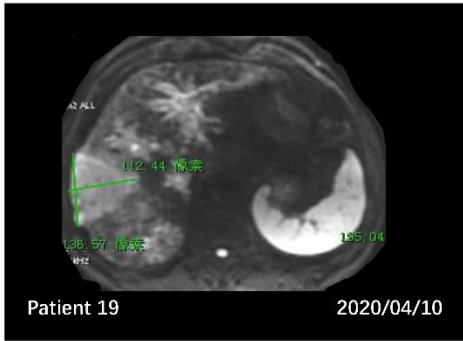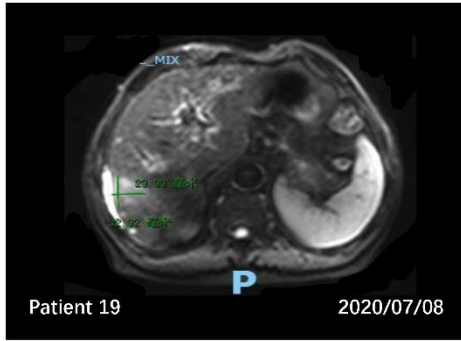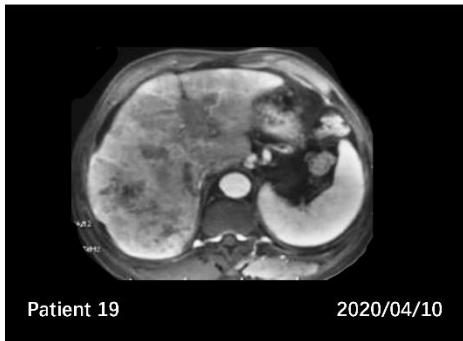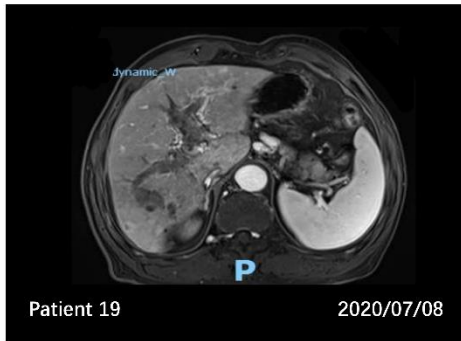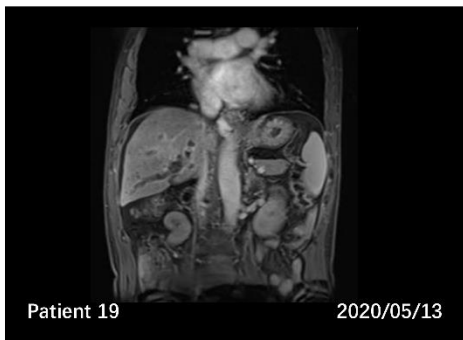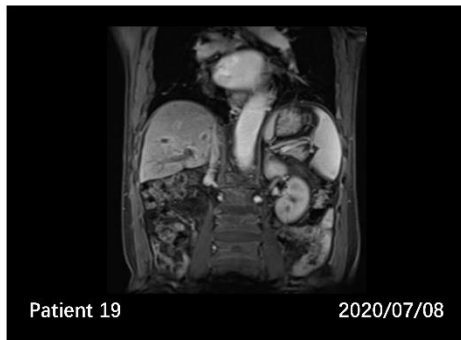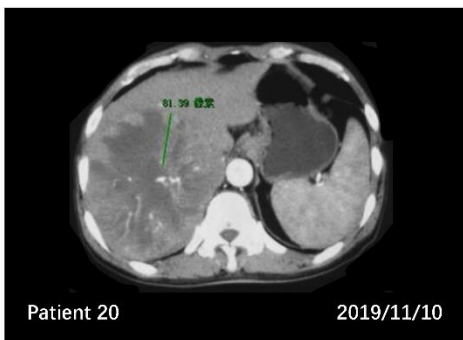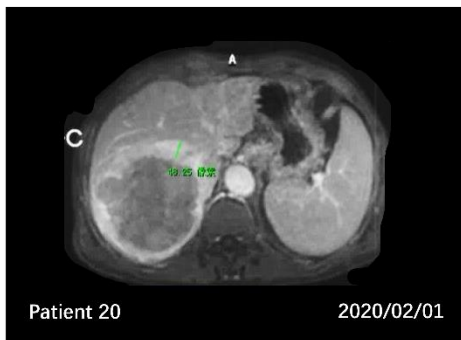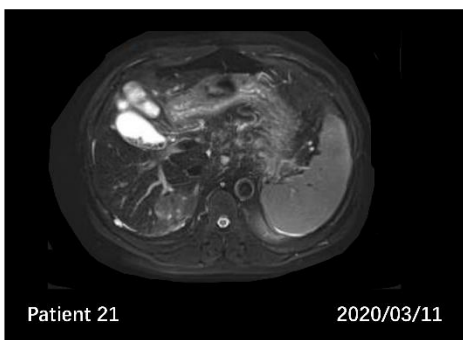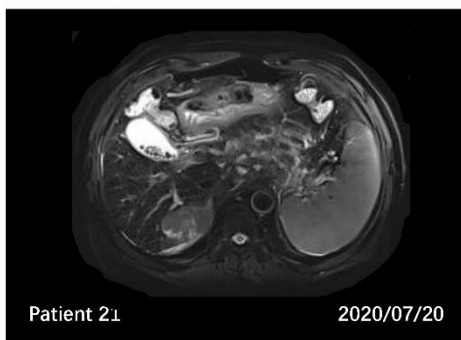

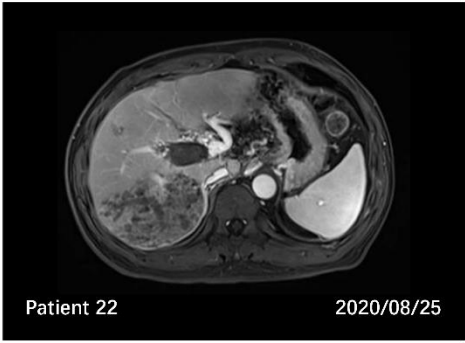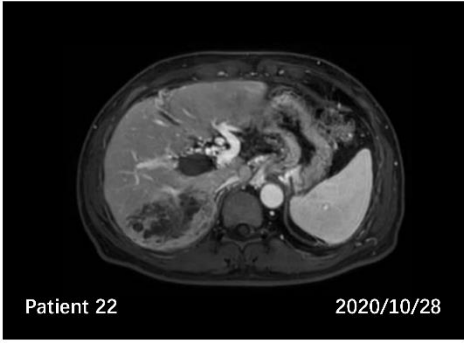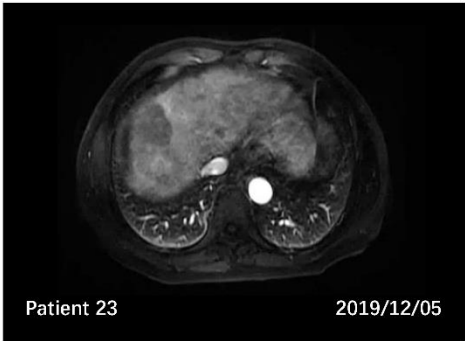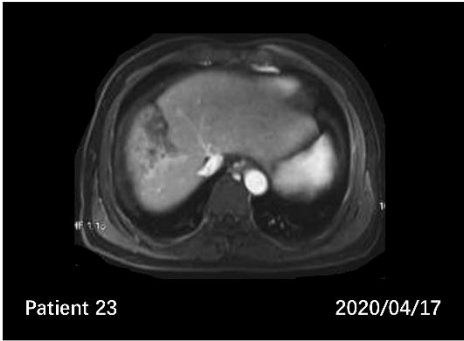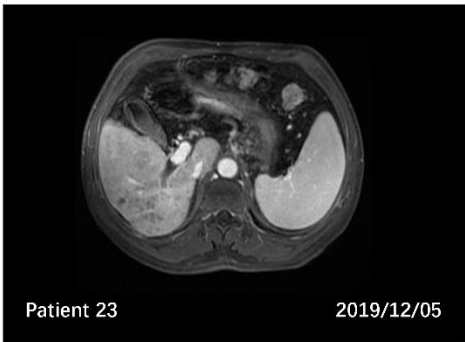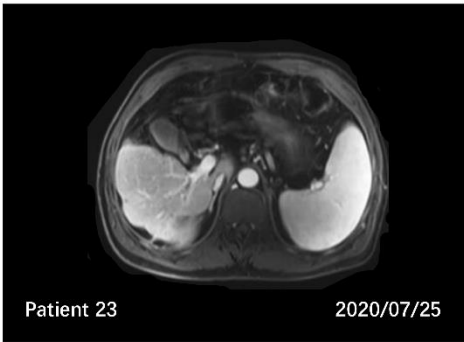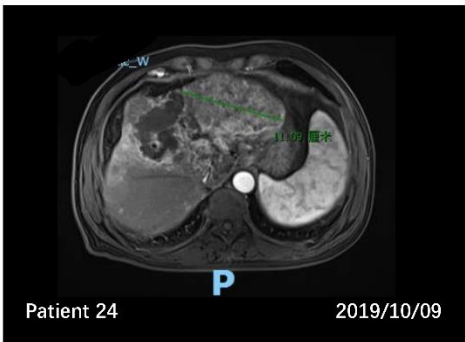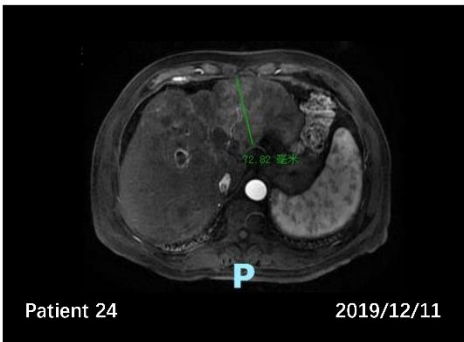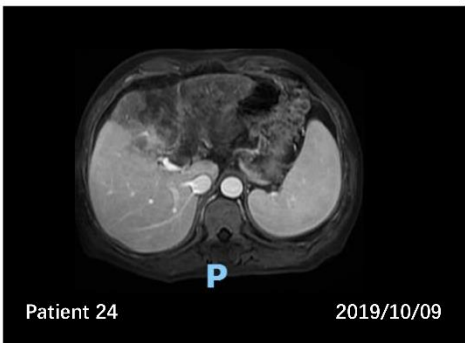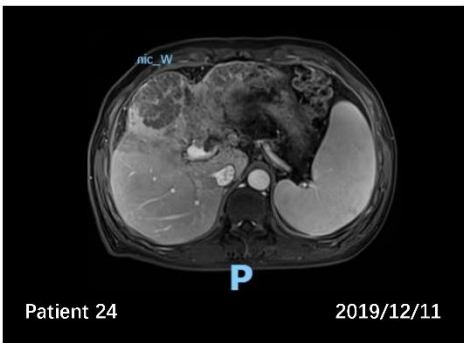

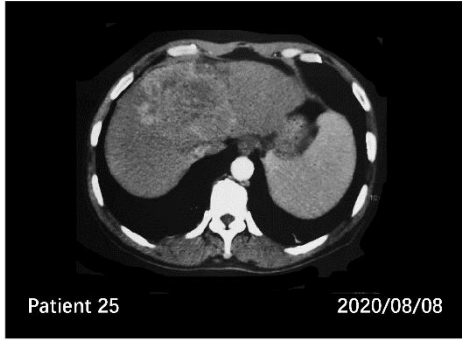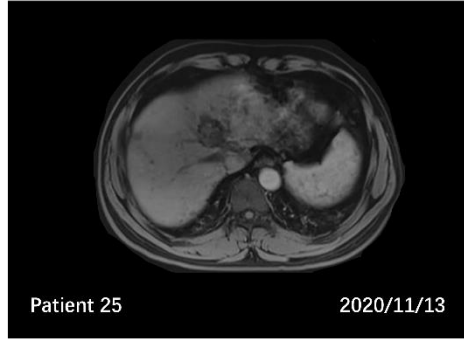

Supplement: Supplementary file 1 [file DataSheet_1.zip › Supplementary Figure 2.PDF]
