# Supplementary material for: Surgical Conversion for Initially Unresectable Locally Advanced Hepatocellular Carcinoma Using a Triple Combination of Angiogenesis Inhibitors, Anti-PD-1 Antibodies, and Hepatic Arterial Infusion Chemotherapy: A Retrospective Study
Source: Front Oncol. 2021 Nov 12;11:729764. doi: 10.3389/fonc.2021.729764 (PMC8632765; doi:10.3389/fonc.2021.729764)
Supplement: Supplementary file 2 [file Table_1.docx]

Supplementary table

**S-Table 1.** Summary of the most common treatment-related adverse events in patients with advanced hepatocellular carcinoma receiving triple therapy (n = 34).

| Preferred AE Term | Any grade | | Grade 1 | | Grade 2 | | Grade 3 | |
| --- | --- | --- | --- | --- | --- | --- | --- | --- |
| Neutropenia | 9 | (26.5) | 3 | (8.8) | 6 | (17.6) | 0 |  |
| Leukopenia | 8 | (23.5) | 2 | (5.9) | 5 | (14.7) | 1 | (2.9) |
| AST level increased | 7 | (20.6) | 2 | (5.9) | 3 | (8.8) | 2 | (5.9) |
| Anemia | 7 | (20.6) | 4 | (11.8) | 3 | (8.8) | 0 |  |
| ALT level increased | 6 | (17.6) | 3 | (8.8) | 2 | (5.9) | 1 | (2.9) |
| Hypoalbuminemia | 6 | (17.6) | 6 | (17.6) | 0 |  | 0 |  |
| Gastrointestinal bleeding | 6 | (17.6) | 2 | (5.9) | 0 |  | 4 | (11.8) |
| Serum bilirubin increase | 5 | (14.7) | 4 | (11.8) | 1 | (2.9) | 0 |  |
| Rash | 5 | (14.7) | 2 | (5.9) | 3 | (8.8) | 0 |  |
| Hypertension | 4 | (11.8) | 0 |  | 3 | (8.8) | 1 | (11.8) |
| Hyperglycemia | 4 | (11.8) | 4 | (11.8) | 0 |  | 0 |  |
| Oulorrhagia | 4 | (11.8) | 3 | (8.8) | 1 | (2.9) | 0 |  |
| Fatigue | 3 | (8.8) | 3 | (8.8) | 0 |  | 0 |  |
| Proteinuria | 3 | (8.8) | 0 |  | 3 | (8.8) | 0 |  |
| Diarrhea | 2 | (5.9) | 2 | (5.9) | 0 |  | 0 |  |
| Nausea | 2 | (5.9) | 2 | (5.9) | 0 |  | 0 |  |
| Pruritus | 2 | (5.9) | 1 | (2.9) | 1 | (2.9) | 0 |  |
| Edema peripheral | 2 | (5.9) | 2 | (5.9) | 0 |  | 0 |  |
| Epistaxis | 2 | (5.9) | 2 | (5.9) | 0 |  | 0 |  |
| Decreased appetite | 2 | (5.9) | 2 | (5.9) | 0 |  | 0 |  |
| Hypothyroidism | 1 | (2.9) | 0 |  | 1 | (2.9) | 0 |  |
| Weight decreased | 1 | (2.9) | 1 | (2.9) | 0 |  | 0 |  |
| Abdominal distention | 1 | (2.9) | 1 | (2.9) | 0 |  | 0 |  |
| Arthralgia | 1 | (2.9) | 1 | (2.9) | 0 |  | 0 |  |
| Gastrohelcoma | 1 | (2.9) | 0 |  | 1 | (2.9) | 0 |  |

AE, adverse event; ALT, alanine aminotransferase; AST, aspartate transaminase
