# Supplementary material for: Surgical Conversion for Initially Unresectable Locally Advanced Hepatocellular Carcinoma Using a Triple Combination of Angiogenesis Inhibitors, Anti-PD-1 Antibodies, and Hepatic Arterial Infusion Chemotherapy: A Retrospective Study
Source: Front Oncol. 2021 Nov 12;11:729764. doi: 10.3389/fonc.2021.729764 (PMC8632765; doi:10.3389/fonc.2021.729764)
Supplement: Supplementary file 3 [file Table_2.docx]

**S-Table 2.** Summary of best response by mRECIST.

| Variable | Patients included in the study  (n = 25) | | Patients treated and evaluated  (n = 32*) | |
| --- | --- | --- | --- | --- |
| Complete response | 12 | (48.0) | 12 | (37.5) |
| Partial response | 12 | (48.0) | 14 | (43.7) |
| Stable disease | 1 | (4.0) | 5 | (15.6) |
| Progressive disease | 0 | (0) | 1 | (3.1) |
| Objective response rate | 24 | (96.0) | 26 | (81.2) |
| Received hepatic resection | 14 | (56.0) | 14 | (43.7) |

*Two of the 34 patients unfinished review assessments.
