# Supplementary material for: Surgical Conversion for Initially Unresectable Locally Advanced Hepatocellular Carcinoma Using a Triple Combination of Angiogenesis Inhibitors, Anti-PD-1 Antibodies, and Hepatic Arterial Infusion Chemotherapy: A Retrospective Study
Source: Front Oncol. 2021 Nov 12;11:729764. doi: 10.3389/fonc.2021.729764 (PMC8632765; doi:10.3389/fonc.2021.729764)
Supplement: Supplementary file 4 [file Table_3.docx]

**S-Table 3.** Efficacy of different drug combinations.

|  | apatinib | | lenvatinib | | sorafenib | | |
| --- | --- | --- | --- | --- | --- | --- | --- |
|  | n = 7 | % | n = 17 | % | n = 1 | % | |
| HR | 3 | 42.86 | 10 | 58.82 | 1 | 100.00 | |
| CR | 4 | 57.14 | 7 | 41.18 | 0 | 0.00 | |
| pCR | 3 | 42.86 | 4 | 23.53 | 1 | 100.00 | |
| PR | 2 | 28.57 | 10 | 58.82 | 1 | 100.00 | |
| SD | 1 | 14.29 | 0 | 0.00 | 0 | 0.00 | |
| ORR | 6 | 85.71 | 17 | 100.00 | 1 | 100.00 | |
|  |  | |  | |  | |  |
|  | camrelizumab | | sintilimab | |  |  |  |
|  | n = 13 | % | n = 12 | % |  |  |  |
| HR | 7 | 53.85 | 7 | 58.33 |  |  |  |
| CR | 6 | 46.15 | 6 | 50.00 |  |  |  |
| pCR | 5 | 38.46 | 2 | 16.67 |  |  |  |
| PR | 6 | 46.15 | 6 | 50.00 |  |  |  |
| SD | 1 | 7.69 | 0 | 0.00 |  |  |  |
| ORR | 12 | 92.31 | 12 | 100.00 |  |  |  |

|  | apatinib + camrelizumab | | | | lenvatinib + camrelizumab | | | sorafenib + camrelizumab | | |  |
| --- | --- | --- | --- | --- | --- | --- | --- | --- | --- | --- | --- |
|  | | n = 5 | | % | | n = 7 | % | n = 1 | % | | |
| HR | | 2 | | 40.00 | | 4 | 57.14 | 1 | 100.00 | | |
| CR | | 2 | | 40.00 | | 3 | 42.86 | 0 | 0.00 | | |
| pCR | | 2 | | 40.00 | | 2 | 28.57 | 1 | 100.00 | | |
| PR | | 2 | | 40.00 | | 4 | 57.14 | 1 | 100.00 | | |
| SD | | 1 | | 20.00 | | 0 | 0.00 | 0 | 0.00 | | |
| ORR | | 4 | | 80.00 | | 7 | 100.00 | 1 | 100.00 | | |
|  | | |  | | |  | |  | |  | |
|  | | | apatinib + sintilimab | | | lenvatinib + sintilimab | |  |  |  |  |
|  | | | n = 2 | % | | n = 10 | % |  |  |  |  |
| HR | | | 1 | 50.00 | | 6 | 60.00 |  |  |  |  |
| CR | | | 2 | 100.00 | | 4 | 40.00 |  |  |  |  |
| pCR | | | 1 | 50.00 | | 1 | 10.00 |  |  |  |  |
| PR | | | 0 | 0.00 | | 6 | 60.00 |  |  |  |  |
| SD | | | 0 | 0.00 | | 0 | 0.00 |  |  |  |  |
| ORR | | | 2 | 100.00 | | 10 | 100.00 |  |  |  |  |

HR, hepatic resection; CR, complete response; pCR, pathologic CR; PR, partial response; SD, stable disease; ORR, objective response rate.
